# Holoprosencephalia, hypoplasia of corpus callosum and cerebral heterotopia in a male belted Galloway heifer with adipsia

**DOI:** 10.1186/s12917-022-03152-4

**Published:** 2022-01-20

**Authors:** Jasmin Nessler, Christian Wunderlich, Deborah Eikelberg, Andreas Beineke, Jonathan Raue, Martin Runge, Andrea Tipold, Martin Ganter, Jürgen Rehage

**Affiliations:** 1 Department for Small Animal Internal Medicine and Surgery, University of Veterinary Medicine Hannover, Foundation, Buenteweg 9, 30559 Hannover, Germany; 2 Clinic for Cattle, University of Veterinary Medicine Hannover, Foundation, Bischofsholer Damm 15, 30173 Hannover, Germany; 3 Institute for Pathology, University of Veterinary Medicine Hannover, Foundation, Buenteweg 17, 30559 Hannover, Germany; 4 Lower Saxony State Office for Consumer Protection and Food Safety Food and Veterinary Institute Braunschweig/Hannover, Eintrachtweg 17, 30173 Hannover, Germany; 5 Clinic for Swine, Small Ruminants and Forensic Medicine, University of Veterinary Medicine Hannover, Foundation, Bischofsholer Damm 15, 30173 Hannover, Germany; 6 Clinic for Cattle, University of Veterinary Medicine Vienna (Vetmeduni Vienna), Veterinaerplatz 1, A-1210 Vienna, Austria

**Keywords:** Thirst, Drinking, Cattle, Neurological disorder, Hypernatremia, Encephalopathy, Malformation

## Abstract

**Background:**

Specialized neurons in the diencephalon detect blood hypernatremia in dehydrated animals. These neurons are connected with the pituitary gland, subsequently producing antidiuretic hormone to reabsorb water from urine in the kidneys, and to the forebrain to generate thirst and trigger drinking behavior.

**Case presentation:**

This is the first case report describing clinical findings, magnetic resonance imaging (MRI) and necropsy results of a Belted Galloway heifer with severe clinical signs of dehydration and hypernatremia, but concurrent adipsia and isosthenuria. Due to insufficient recovery with symptomatic treatment, owners elected euthanasia. Postmortem MRI and necropsy revealed a complex forebrain malformation: mild abnormal gyrification of the forebrain cortex, lobar holoprosencephaly, and corpus callosum hypoplasia. The affected brain structures are well known to be involved in osmoregulation and generation of thirst in dogs, humans and rodents.

**Conclusions:**

Complex forebrain malformation can be involved in the pathogenesis of hypernatremia and adipsia in bovines.

## Background

Thirst is an essential motivation to seek and drink water for all mammals to survive [1]. Water uptake is essential to stabilize the equilibrium of osmolality within body fluids. Several mechanisms ensure, that a mammal is well hydrated [1]. Decreased amount of water in the body leads to increased blood osmolality and decreased extracellular fluid volume [2]. Several mechanisms are known to detect and correct dehydration [1]. Amongst others, baroreceptors in the kidney detect decreased blood pressure and lead to production of angiotensin II. Specialized neurons in the *lamina terminalis* of the diencephalon that lay outside the blood-brain-barrier detect changes in osmolality, for example increased sodium blood content, and increased angiotensin II [1, 3–5]. This leads to increased release of antidiuretic hormone (ADH) from the pituitary gland into the blood [4, 6]. ADH leads to retention of water in the kidneys, subsequently plasma dilution and on the same time urine concentration (hypersthenuria) [1]. Additionally, the *lamina terminalis*, a midline structure ventral of the corpus callosum, projects into several regions of the brain that generate the feeling of thirst. Amongst others, therefore an important region is located in the cingulate gyrus dorso-lateral of the corpus callosum [7]. Summarizing, when the body detects dehydration, it starts to save water as it concentrates the urine and tries to supply water by stimulating the mammal to drink. These mechanisms seem comparable in monogastric animals and ruminants [1, 3–6]. Pathological absence of drinking behavior is named adipsia [8]. In the following case report we show clinical, laboratory and magnetic resonance imaging (MRI) findings of a heifer with a congenital brain malformation that interferes with generation of thirst and the equilibrium of osmolality.

## Case presentation

A 9.5 month old male Belted Galloway heifer was presented in the Clinic for Cattle of the University of Veterinary Medicine Hannover, Foundation due to a 6 month old fistulating neck injury and progressive behavioral changes. The animal was kept in a non-commercial herd under pasture feeding conditions with a free available shelter. In the winter, hay was supplemented and water was available ad libitum. Additionally carrots and apples were fed to keep the animals hand-tame. The owners reported that the heifer separated from the herd and seemed inattentive in the last months.

All examinations were performed with written informed owner’s consent in accordance with the University’s ethical guidelines.

Clinical examinations were performed by and under supervision of a Diplomat of the European College of Bovine Health Management and by a Diplomat and a Resident of the European College of Veterinary Neurology.

Blood examinations of venous blood samples were performed from samples of the jugular or tail vein. Urinary analysis was performed from spontaneous urine samples. Fractional excretion (FE) of electrolytes shows the percentage of electrolytes filtered by the kidney into the urine. Therefore the electrolytes in plasma and urinary at the same time point were related to glomerular filtration rate calculated using creatinine filtration. FE was calculated with the formula $FE\ electrolyt=\frac{\left( electrolyt\ urine\ast creatinine\ plasma\right)}{\left( electolyt\ plasma\ast creatinine\ urine\right)}\ast 100$ [9].

Radiography (FDR Go Flex Vet, Fujifilm, Tokio, Japan) of the neck in latero-lateral plane, ultrasonographic examination (Sonovet 2000, Medison, Soul, Korea) of kidneys, sampling of kidney biopsy via true cut and lumbar cerebrospinal fluid sampling were performed in standing position. The latter were performed after regional anesthesia.

MRI (3.0 T MRI scanner Achieva, Philips Medical Systems, Best, The Netherlands; coil: SENSE-NV-16) was performed within 1 hour postmortem of the head and the cervical spine until the fourth cervical vertebra without any further tissue fixation. The protocol included a T2-weighted (T2w) fast spin echo sequence in sagittal, dorsal and transversal plane of the head, a sagittal plane of the cervical spinal cord (repetition time [TR] 3000 ms, echo time [TE] 13 ms), a fluid attenuated inversion recovery sequence (FLAIR) of the head in transversal plane (TR 10,000 ms, TE 36 ms), a T1-weighted (T1w) 3D gradient echo sequence of the head (TR 11.432 ms, TE 2.2 ms) with reconstruction of a dorsal, a sagittal and a transversal plane.

Necropsy of the full body was performed. Gross examination of the whole brain was performed before formalin fixation and macroscopical evaluation was performed after formalin fixation and transversal cutting. Microscopical evaluation was performed after hematoxylin-eosin-staining of representative samples of grey and white matter of the forebrain, brainstem and cerebellum as well as pituitary gland [10]. Bielschowsky stain and immunohistochemical staining against glial fibrillary acidic protein (GFAP) of caudate nucleus, corpus callosum and brainstem were performed as previously described [11, 12]. Anatomy of the heifer’s brain structures in MRI and necropsy was subjectively compared to normal anatomy [13, 14].

On clinical examination the heifer was cachectic, appeared dull and was inappetent. During hospitalization the heifer did not drink water. On the left side in the cranial third of the neck the heifer showed a subdermal firm mass with approximately 5 cm in diameter, which felt adherent to the trachea. A fistula canal reached from the skin into the mass. The wound showed mild sero-purulent secretion. Generalized markedly reduced skin turgor and bilateral enophthalmus were present. Mucous membranes felt sticky. On rectal examination the feces were dry and hard. Estimated dehydration was mild to moderate (5–8% water loss). Neurological examination revealed mild obtundation, compulsive pacing, and mild proprioceptive ataxia especially of the pelvic limbs. Menace response was decreased bilaterally. Other cranial nerve examinations and spinal reflexes were normal. A diffuse forebrain pathology was suspected.

Radiographic examination of the area of the mass at the neck showed a focal well delineated spot of mildly decreased radiodensity within the neck parenchyma, which was suspected to be gas. Blood examination revealed mild leukopenia, elevated hematocrit, elevated gamma glutamyltransferase activity, severe increased urea and creatinine levels, hyperphosphatemia and severe hypernatremia, hyperkalemia and hyperchloremia, as well as mild hyposelenemia (Table 1). Urine was isosthenuric despite high levels of serum sodium and signs of dehydration: specific gravity was 1024. FE was normal for sodium, calcium, and phosphor, which indicated normal renal function. Only increased FE of potassium was indicative of inappropriate renal loss (Table 2). Ultrasonographic examination of kidneys and histologic examination of kidney biopsy showed no abnormalities. Serum samples were negatively tested for bovine viral diarrhea and bovine herpes virus infection 1 using standard cell culture procedures for routine diagnostics in the Food and Veterinary Institute Braunschweig/Hannover. Examination of lumbosacral samples of cerebrospinal fluid was unremarkable.

**Table 1:** Laboratory parameters of the heifer

Parameter	hospitalization	seven days after treatment	reference values for adult cows
Leukocytes per μl	6400	11,200	8000-10,000/μl
Erythrocytes * 10^6^/μl	8.39	4.9	6.0–8.0 * 10^6^/μl
Hemoglobin in g/dl	12.0	7.0	8.0–14.0 g/dl
Hematocrit in %	43.1	23.2	25.0–35.0%
MCV in μm^3^	51.4	47.3	40.0–60.0 μm^3^
MCH in pg	14.3	14.3	14.0–20.0 pg
MCHC in g/dl	27.8	30.2	26.0–34.0 g/dl
Platelets per μl	174,000	447,000	200,000-800,000/ μl
Total protein in g/l	68	57	60.0–80.0 g/l
Total bilirubin in μmol/l	3.8	0.9	< 7.0 μmol/l
AST in U/l	117	246	< 100 U/l
GGT in U/l	76	45	< 33 U/l
GLDH in U/l	9.8	126	< 14 U/l
Cholesterol in mmol/l	6.88	1.72	> 3.0 mmol/l
Urea in mmol/l	21	4.77	< 8.0 mmol/l
Creatinin in μmol/l	323	123	< 150 μmol/l
Albumin in g/l	35.8	33.3	30.0–40.0 g/l
Calcium in mmol/l	2.6	2.33	2.1–3.0 mmol/l
Magnesium in mmol/l	0.94	0.73	0.7–1.2 mmol/l
Phosphorus in mmol/l	3.16	0.93	1.1–2.4 mmol/l
Selenium in μg/l	66	–	> 75 μg/l
Sodium in mmol/l	183	153	135–145 mmol/l
Potassium in mmol/l	5.72	3.45	3.5–4.5 mmol/l
Chloride in mmol/l	139	115	90–110 mmol/l
Glucose in mmol/l	4.57	–	3.0–3.9 mmol/l

Laboratory parameters of the heifer at the beginning of hospitalization and after seven days continuous rate infusion with hypotonic fluid and enforced oral fluid uptake; reference from laboratory given for adult cattle

*MCV* mean corpuscular volume, *MCH* mean corpuscular hemoglobin, *MCHC* mean corpuscular/cellular hemoglobin concentration, *AST* aspartate aminotransferase activity, *GGT* gamma glutamyltransferase activity, *GLDH* glutamate dehydrogenase activity, *U* units

**Table 2:** Laboratory parameters of the heifer, renal excretion of electrolytes

	plasma [mmol/l]	urine [mmol/l]	FE [%]	FE [%] reference in calves [7]
creatinine	0.139	14.39		
sodium	155.5	140.5	0.87	0.0–2.7
calcium	2.49	0.78	0.3	0.0–2.2
phosphor	0.84	3.39	4.22	2.7–23.6
potassium	3.47	364.5	94.14	15–85

Increased fractional excretion (FE) is a hint for increased elimination of electrolytes due to renal failure

$$FE\;electrolyt=\frac{\left(electrolyt\;urine\ast creatinine\;plasma\right)}{\left(electolyt\;plasma\ast creatinine\;urine\right)}\ast100$$

Summarizing, a fistulation injury at the neck, which was considered clinically irrelevant, signs of a diffuse forebrain lesion, adipsia despite clinically evident hypovolemia and hypernatremia and for this condition inappropriate isosthenuria were found. Additionally, prerenal azotemia due to dehydration, adipsic central diabetes insipidus, and beginning of a secondary nephropathy were suspected.

The heifer was slowly rehydrated with continuous rate infusion of hypotonic fluid (6 ml/kg body weight per day intravenous isotonic saline solution (“Isotonische Natriumchlorid-Lösung ad us. vet.”, Bela-Pharm GmbH & Co. KG, Vechta, Germany) with 2% of 40% glucose solution (“Glucose-Lösung 40% ad us. vet.”, Bela-Pharm GmbH & Co. KG)) and enforced oral fluid uptake (water, 50 ml/ kg body weight per day per os). Plasma sodium concentrations were monitored every 12 to 24 h. Urea and creatinine levels were normal after 7 days (Table 1).

Plasma sodium levels ameliorated, but did not decrease to normal values. Consciousness improved slightly, the heifer started to eat again, but did not drink. Intensive endeavor to motivate the heifer to drink water, e.g. with a suckling bucket, sweetened water or even soaked hay in water, did not result in further clinical improvement. Therefore, the owners elected human euthanasia.

**Fig. 1:**
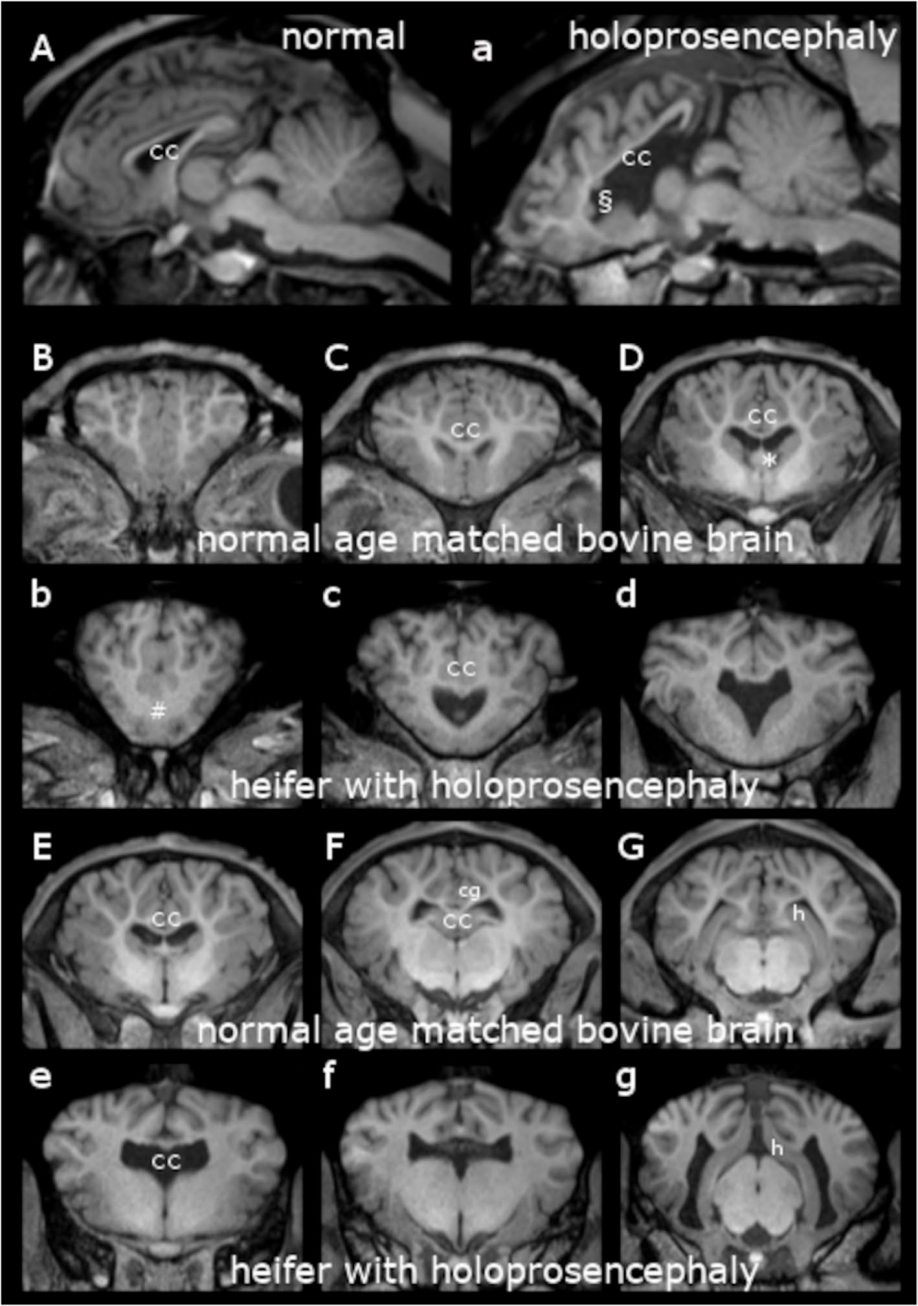
Magnetic resonance imaging (MRI) of a heifer with holoprosencephaly and corpus callosum hypoplasia. T1weighted (T1w) images in midsagittal (A, a) and transversal (B, b-G, g) planes on the level of the frontal lobe (B, b), the genu of corpus callosum (C, c), of the pallidal globe rostral (D, d) and caudal (E, e), of interthalamic adhesion (F, f) and on the level of the geniculate bodies (G, g). A-G: MRI of a normal brain of a age matched Holstein Friesian (1.5 months old, male). The animal showed no symptoms of a forebrain lesion and underwent routine in vivo diagnostic imaging due to a disease unrelated to encephalopathy. a-g: MRI of a heifer with holoprosencephaly and corpus callosum hypoplasia. Note the incomplete separation of both cerebral hemispheres at the frontal lobe (b”#”), the generalized abnormal gyrification of the cerebral cortex including the cingulate gyrus (normal: F “cg”; absent in f), absent septum pellucidum and (“*” in D, absent in d) and corpus callosum (“cc”) with decreased thickness and altered shape. The hippocampal tail (G and g “h”) seemed deviated dorsally into the *sulcus corporis callosi*. Additionally cerebral cortex heterotopia is suspected (“§” in a). Both lateral and the third ventricle were enlarged without any signs of elevated intracranial pressure (a, c-g)

MRI revealed a subjective size reduction or complete absence of most cerebral midline structures (Fig. 1): septum pellucidum, septal nuclei, paraterminal gyrus and the fornix were not visible while the pallidal globe, cingulate gyrus and corpus callosum were decreased in size. Subsequently both cerebral hemispheres were not properly separated. In consequence, hippocampal structures were not clearly to distinguish. Additionally, abnormal gyrification was visible within the whole cerebral cortex. The hippocampal tail was deviated dorsally into the *sulcus corporis callosi*. Two small focal, well demarcated areas of grey matter were visible adjacent to the lateral ventricles, where physiologically no grey matter should be located. This was suspected to be nodular heterotopic cerebral cortex (Fig. 1, a§). Both lateral and the third ventricle were enlarged and misshaped without any signs of elevated intracranial pressure. Pituitary gland was 8 mm of height with normal signal intensity and hypothalamus seemed normal. No altered signal intensity in CNS parenchyma indicative of a cerebral edema or pontine myelinolysis was visible. Lobar holoprosencephaly with partial corpus callosum hypoplasia, abnormal cerebral gyrification and focal cortical dysplasia was suspected.

MRI of the cranial cervical region showed a subdermal lobulated mass lesion, well demarcated, 2.5 cm in diameter, hyperintense to surrounding muscles, adjacent to the latero-ventral aspect of the larynx with contact to the skin surface.

**Fig. 2:**
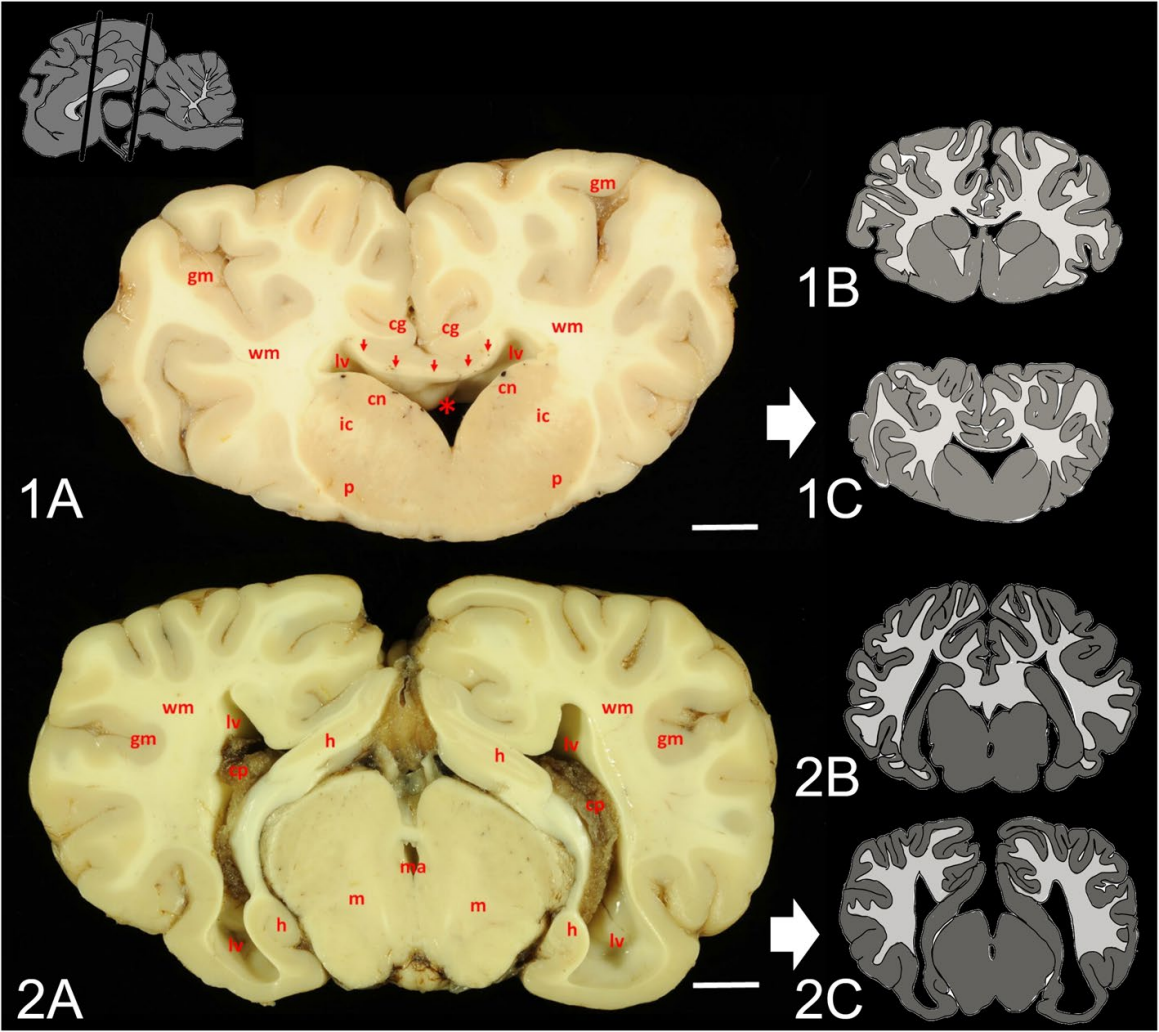
Necropsy finding of a heifer with holoprosencephaly and corpus callosum hypoplasia. 1A) Cross section of the brain at the level of the pallidal globe: The septum pellucidum (asterisk) is absent. The corpus callosum (arrows) and caudate nucleus (cn) are reduced in size and gyri and sulci of the cerebral hemispheres are flattened. Cerebral grey matter (gm), cerebral white matter (wm), cingulate gyrus (cg), internal capsule (ic), lateral ventricle (lv), putamen (p), scale bar = 1 cm. 1B) Schematic picture of a normal brain at the same level. 1C) Schematic picture of the affected heifer’s brain on the same level. 2A) Cross section of the brain at the level of the lateral geniculate body: Note dilated lateral ventricles (lv) and flattened gyri and sulci of the cerebral hemispheres. Cerebral grey matter (gm), cerebral white matter (wm), choroid plexus (cp), hippocampus (h), mesencephalic aqueduct (ma), mesencephalon (m), scale bar = 1 cm. 2B) Schematic picture of a normal brain at the same level. 2C) Schematic picture of the affected heifer’s brain on the same level. Level of transverse sections are given in the upper schematic sagittal picture of a normal brain. Schematic pictures of normal brain modified after Schmidt et al. [14]

**Fig. 3:**
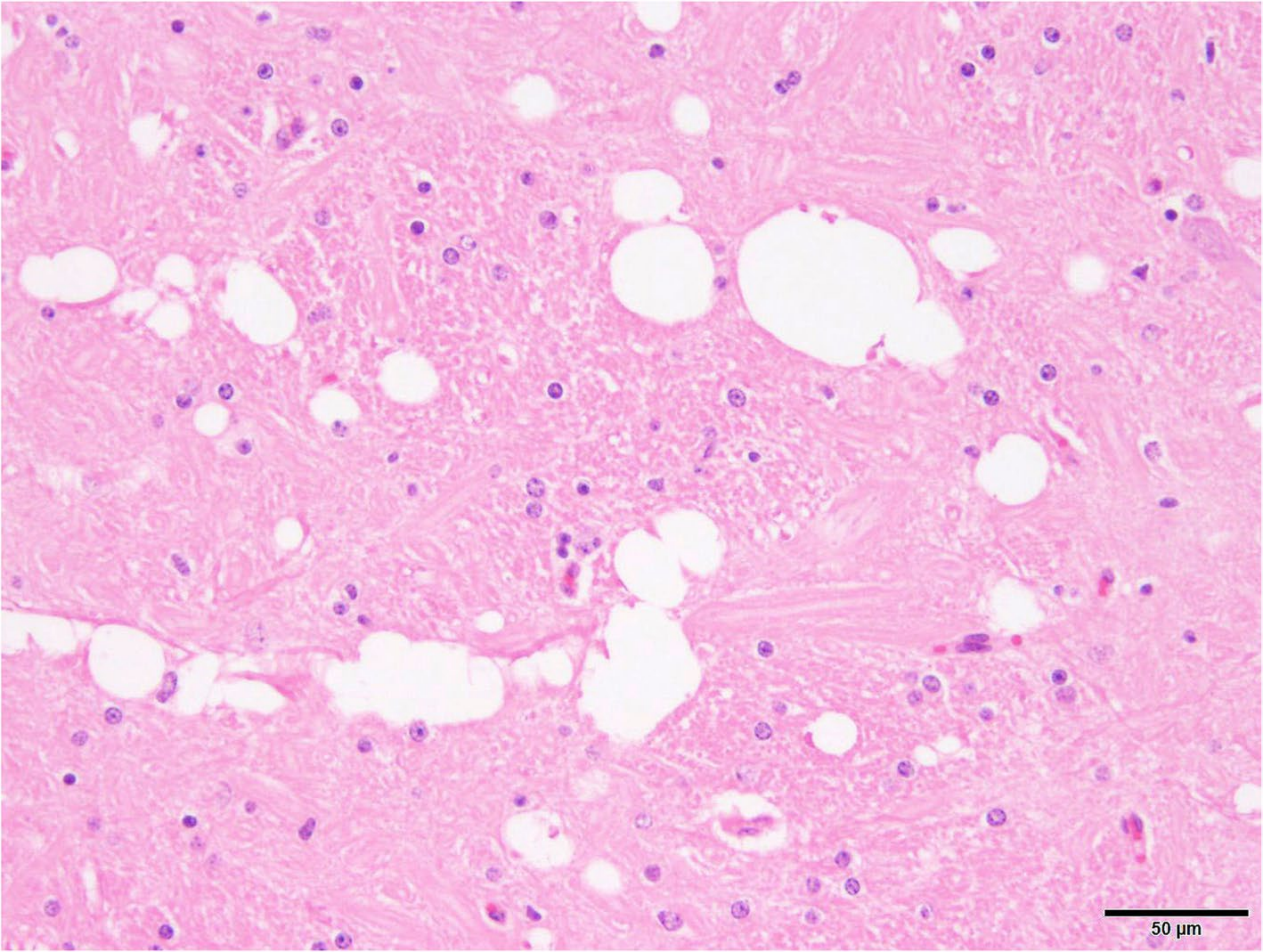
Histopathology white matter. Vacuolization of the neuroparenchmyma. Hematoxylin eosin-staining; magnification 400x

Gross examination of the brain supported MRI results in pathology (Fig. 2): The septum pellucidum was absent and the lateral ventricles moderately enlarged in size. The corpus callosum and caudate nucleus were reduced in size and gyri and sulci of the cerebral hemispheres were flattened. Pituitary gland was macroscopically normal. Histology of hematoxylin eosin-stained section revealed the presence of mild vacuolization of the hippocampal neuroparenchyma and cerebellar white matter (Fig. 3). Corpus callosum showed loss of its physiological linear structure. Special stainings neither showed astrogliosis nor neurofibrillary tangles.

## Discussion and conclusions

In this case report, we present a heifer with hypovolemic hypernatremia and adipsia associated with a brain malformation – lobar holoprosencephaly with partial corpus callosum hypoplasia, abnormal cerebral gyrification and focal cortical dysplasia.

Holoprosencephaly is a congenital brain malformation characterized by incomplete midline separation of the prosencephalon into right and left hemispheres and is often accompanied with corpus callosum hypoplasia, abnormal gyri and sulci, and ectopic cerebral grey matter [3]. Holoprosencephaly is divided in three subtypes: lobar holoprosencephaly, where the right and left ventricles are separated, but some midline structures - like the septum pellucidum or corpus callosum - are not present or at least hypoplastic; semilobar holoprosencephaly with partial separation; and the most severe form, alobar holoprosencephaly, with one single brain ventricle and no interhemispheric fissure [15]. In the described heifer, lobar holoprosencephaly was diagnosed, as dorsal interhemispheric fissure is present, but rostro-ventral separation of the prosencephalon is incomplete and corpus callosum and septum pellucidum are reduced in size or absent. Other neuronal migration disorders might be associated with holoprosencephaly [16]; e.g. focal cerebral heterotopia, which are ectopic neurons that failed to migrate to their designated anatomical region and instead build grey matter at any point of their initial migration route [16]. Nodular heterotopia mostly occurs as little grey matter nests in the telencephalon, in humans mostly in the subependymal zone or just below the neocortex [16].

Holoprosencephaly occurs, if embryological dorsal and/or ventral patterning of the brain is disturbed that takes place between day 18 and 28 of gestation in humans. Especially disturbance of Sonic hedgehog (Shh) or bone morphogenetic protein (BMP) signaling pathways seems to be involved [17, 18], either by insufficient production of Shh in the notochord or by other disturbance of the signaling pathway [18]. This might be the case in genetic defects known in humans, mice and zebrafish [19–22], due to environmental or metabolic influences at time of gestation, like exposure of the fetus to toxins (e.g. alcohol, *Veratrum californicum* intoxication, and others [19, 23]), maternal diabetes mellitus [24], or inborn errors of metabolism [25, 26], or due to intrauterine viral infections [27, 28]. In the case of the described heifer, the aetiology remains unknown. Bovine viral diarrhea virus, well known for causing intraurine infection and malformation in bovines [29], was not found.

Corpus callosum aplasia and holoprosencephaly are well described in large animals [30–35], but adipsia has not been reported so far. If the body does not contain enough water, concentration of sodium and osmolality in the blood raise [8]. Additionally, due to decreased blood volume, blood pressure drops (=hypotension). Baroreceptors in the kidney inter alia detect hypotension [4]. Therefore, the kidneys produce renin [1]. Renin converts angiotensin I into angiotensin II, which leads to vasoconstriction and increased heart rate, which will increase blood pressure [1]. In a physiological brain, osmoreceptors in the diencephalon detect the increased amount of sodium and angiotensin II in the blood, as they are located in the *lamina terminalis* outside of the blood brain barrier. Efferent fibers stimulate the pituitary gland to produce antidiuretic hormone (ADH) [1, 3, 4]. ADH will lead to increased reabsorption of water in the kidney [1]. This reduces urine volume and increases urine specific gravity while diluting plasma. Additionally, efferent fiber of the *lamina terminalis* reach in different areas of the brain to generate thirst [1, 4]. Amongst others, it was shown that the cingulate gyrus is active, if a human gets thirsty [1]. This leads to increased water intake via drinking. Understanding this mechanism is important to diagnose the cause for hypernatremia in the heifer: In mammals with pathologically high sodium in the blood (=hypernatremia) several differential diagnosis are feasible. There might be an increased intake of sodium per os or parenterally, e.g. salt intoxication or iatrogenic infusion with hypertonic saline solution. In this scenario the animal will try to compensate increased osmolality by increased reabsorption of water in the kidneys, which will lead to hypersthenuria (=increased concentration of urine), and by drinking, if possible [2]. In ruminants, additional the rumen serves as water reservoir, where up to 49% of total body fluid can be stored [36]. In the ruminal wall tight junctions and active transporters regulate water and sodium transition into the blood [37] and allow a very high osmotic gradient [36] between plasma and rumen. This way, short-term water shortage can be bridged. ADH-independent mechanism additionally lead to massively decreased salivation with an increased saliva-sodium-content to redistribute sodium from the plasma into the rumen to decrease plasma-rumen-sodium-gradient [36]. Unfortunately, saliva sodium was not measured in this case report.

Hypernatremia can also be caused by loss of free water mostly via the kidneys (e.g. in central or renal diabetes insipidus or renal failure). In the heifer, normal fractional excretion of most electrolytes did not indicate tubular malfunction [9]. Loss of free water will lead to high amount of urine, less concentrated urine and decreased specific urinary gravity (=hyposthenuria) [2]. But, if the animal has access to water, it will compensate its fluid loss by increased drinking. This is called to polyuria and polydipsia (PU/PD) [8].

The third differential diagnosis is water deprivation. Sodium concentration in the blood will raise and the kidneys will reabsorb water from the urine, which leads to hypersthenuria [2].

In the present case the heifer showed severe signs of dehydration and hypernatremia without any visible attempt of the body to compensate the fluid deficiency: Neither did the kidneys reinforce water reabsorption from urine – the urine remained isosthenuric - nor did the heifer drink at all. Adipsia and hypernatremia are common signs of a diencephalic lesion in humans and small animals [38–41]. Several etiologies are reported to cause damage to the structures that contain osmoreceptors and generate thirst: Meningoencephalitis of unknown origin, trauma, neoplasia or anomalies [2, 38, 42, 43]. The most common anomaly in dogs causing adipsia is a defect in the midline structures, such as corpus callosum aplasia or hypoplasia and holoprosencephaly [2, 38]. Solitaire corpus callosum abnormalities are mostly asymptomatic in humans or patients only show mild impairment in learning [43]. Solitaire corpus callosum hypoplasia seems rare in veterinary medicine [38]. The majority of reported cases include additional malformations [38]. It seems that in most cases additional structures affected by malformation may cause clinical signs such as mental retardation, behavioral abnormalities, tremor, proprioceptive deficits, ataxia, reduced menace response, obtundation, circling, head tilt and nystagmus, seizures, adipsia, and hypernatremia [38, 43]. In the heifer’s case, postmortem MRI and necropsy confirmed malformation in several forebrain regions: Abnormal diencephalic structures like absent septum pellucidum and abnormal gyrification especially in the cingulate gyrus might have caused hypernatremia and adipsia. Although palliative therapy is possible in dogs [2, 38], significant clinical improvement could not be obtained in the heifer with enforced water intake. It might be, that hypernatremia was too severe to treat it with enforced water intake alone or that neurological signs might be due to the brain malformation rather than from electrolyte disturbance. In veterinary and human medicine severe hypernatremia is a negative prognostic marker in several diseases [44, 45]. Therapy with vasopressin is described for small animals [2], a treatment not legally permitted in food producing animals in Germany.

Summarizing, signs of dehydration and hypernatremia in the context of isosthenuria and adipsia is highly suspicious for disturbance of central osmoregulation in bovine patients. In young animals, anomaly of cerebral midline structures like holoprosencephaly and corpus callosum agenesis should be considered.

## Data Availability

The datasets used and/or analyzed during the current study are available from the corresponding author on reasonable request.

## Abbreviations

ADH: Antidiuretic hormone
AST: Aspartate aminotransferase activity
BMP: Bone morphogenic protein
BVDV: Bovine virus diarrhea virus
FE: Fractional excretion
FLAIR: Fluid attenuated inversion recovery sequence
GGT: Gamma glutamyltransferase activity
GLDH: Glutamate dehydrogenase activity
MCH: Mean corpuscular hemoglobin
MCHC: Mean corpuscular/cellular hemoglobin concentration
MCV: Mean corpuscular volume
MRI: Magnetic resonance imaging
ms: Millisecond
Shh: Sonic hedgehog protein
T1w: T1-weighted
T2w: T2-weighted
TE: Echo time
TR: Repetition time
U: Units
